# Cayley Bipolar Fuzzy Graphs

**DOI:** 10.1155/2013/156786

**Published:** 2013-12-12

**Authors:** Noura O. Alshehri, Muhammad Akram

**Affiliations:** ^1^Department of Mathematics, Faculty of Sciences (Girls), King Abdulaziz University, Jeddah, Saudi Arabia; ^2^Punjab University College of Information Technology, University of the Punjab, Old Campus, Lahore, Pakistan

## Abstract

We introduce the concept of Cayley bipolar fuzzy graphs and investigate some of their properties. We present some interesting properties of bipolar fuzzy graphs in terms of algebraic structures. We also discuss connectedness in Cayley bipolar fuzzy graphs.

## 1. Introduction

Graph theory is an extremely useful tool in solving the combinatorial problems in different areas. Point-to-point interconnection networks for parallel and distributed systems are usually modeled by *directed graphs* (or digraphs). A digraph is a graph whose edges have direction and are called *arcs* (edges). Arrows on the arcs are used to encode the directional information: an arc from vertex (node) *x* to vertex *y* indicates that one may move from *x* to *y* but not from *y* to *x*. The Cayley graph was first considered for finite groups by Cayley in 1878. Max Dehn in his unpublished lectures on group theory from 1909 to 1910 reintroduced Cayley graphs under the name Gruppenbild (group diagram), which led to the geometric group theory of today. His most important application was the solution of the word problem for the fundamental group of surfaces with genus, which is equivalent to the topological problem of deciding which closed curves on the surface contract to a point [1].

The notion of fuzzy sets was introduced by Zadeh [2] as a method of representing uncertainty and vagueness. Since then, the theory of fuzzy sets has become a vigorous area of research in different disciplines. In 1994, Zhang [3] initiated the concept of bipolar fuzzy sets as a generalization of fuzzy sets [4]. A bipolar fuzzy set is an extension of fuzzy sets whose membership degree range is [−1,1]. In a bipolar fuzzy set, the membership degree 0 of an element means that the element is irrelevant to the corresponding property, the membership degree (0,1] of an element indicates that the element somewhat satisfies the property, and the membership degree [−1,0) of an element indicates that the element somewhat satisfies the implicit counterproperty.

Kaufmann's initial definition of a fuzzy graph [5] was based on Zadeh's fuzzy relations [2]. Mordeson and Nair [6] introduced the fuzzy analogue of several basic graph-theoretic concepts. Kauffman [5] defined the concept of complement of fuzzy graph and studied some operations on fuzzy graphs. Akram and Dudek [7–9] introduced many new concepts, including bipolar fuzzy graphs, complete bipolar fuzzy graphs, regular bipolar fuzzy graphs, and irregular bipolar fuzzy graphs. Wu [4] discussed fuzzy digraphs. Shahzamanian et al. [10] introduced the notion of roughness in Cayley graphs and investigated several properties. Namboothiri et al. [11] discussed Cayley fuzzy graphs. In this paper, we introduce the concept of Cayley bipolar fuzzy graphs and investigate some of their properties. We present some interesting properties of bipolar fuzzy graphs in terms of algebraic structures. We also discuss connectedness in Cayley bipolar fuzzy graphs.

## 2. Preliminaries

A digraph is a pair D* = (*V*, *E*), where *V* is a finite set and *E*⊆*V* × *V*. Let *D*_1_* = (*V*_1_, *E*_1_) and *D*_2_* = (*V*_2_, *E*_2_) be two digraphs. The *Cartesian product* of *D*_1_* and *D*_2_* gives a digraph *D*_1_* × *D*_2_* = (*V*, *E*) with *V* = *V*_1_ × *V*_2_ and

(1) E={(x,x2)→(x,y2) ∣ x∈V1,x2→y2∈E2}  ∪{(x1,z)→(y1,z) ∣ x1→y1∈E1,z∈V2}.

In this paper, we will write *xy* ∈ *E* to mean *x* → *y* ∈ *E*, and if *e* = *xy* ∈ *E*, we say *x* and *y* are *adjacent* such that *x* is a starting node and *y* is an ending node.

The study of vertex transitive graphs has a long and rich history in discrete mathematics. Prominent examples of vertex transitive graphs are Cayley graphs which are important in both theory and applications; for example, Cayley graphs are good models for interconnection networks.

**Definition 1**

Let *G* be a finite group and let *S* be a minimal generating set of *G*. A Cayley graph (*G*, *S*) has elements of *G* as its vertices; the edge set is given by {(*g*, *g*_*s*_) : *g* ∈ *G*, *s* ∈ *S*}. Two vertices *g*_1_ and *g*_2_ are adjacent if *g*_2_ = *g*_1_ · *s*, where *s* ∈ *S*. Note that a generating set *S* is minimal if *S* generates *G* but no proper subset of *S* does.

**Theorem 2**

All Cayley graphs are vertex transitive.

**Definition 3**

Let (*V*, ∗) be a group and let *A* be any subset of *V*. Then the Cayley graph induced by (*V*, ∗, *A*) is the graph *G* = (*V*, *R*), where *R* = {(*x*, *y*) : *x* ^−1^ *y* ∈ *A*}.

**Definition 4 (see [2])**

A fuzzy subset *μ* on a set *X* is a map *μ* : *X* → [0,1]. A *fuzzy binary relation* on *X* is a fuzzy subset *μ* on *X* × *X*. By a fuzzy relation we mean a fuzzy binary relation given by *μ* : *X* × *X* → [0,1].

**Definition 5 (see [11])**

Let (*V*, ∗) be a group and let *μ* be a fuzzy subset of *V*. Then the fuzzy relation *R* on *V* defined by

(2) $$\begin{array}{c} R\left(x,y\right)=\left\{\mu \left(x^{-1}*y\right)\forall x,y\in V\right\} \end{array}$$

induces a fuzzy graph *G* = (*V*, *R*), called the *Cayley fuzzy graph* induced by the (*V*, ∗, *μ*).

**Definition 6 (see [3])**

Let *X* be a nonempty set. A *bipolar fuzzy set B* in *X* is an object having the form

(3) $$\begin{array}{c} B=\left\{\left(x,\mu_{B}^{P}\left(x\right),\mu_{B}^{N}\left(x\right)\right)\;|\;x\in X\right\}, \end{array}$$

where *μ*_*B*_^*P*^ : *X* → [0, 1] and *μ*_*B*_^*N*^ : *X* → [−1, 0] are mappings.

We use the positive membership degree *μ*_*B*_^*P*^(*x*) to denote the satisfaction degree of an element *x* to the property corresponding to a bipolar fuzzy set *B* and the negative membership degree *μ*_*B*_^*N*^(*x*) to denote the satisfaction degree of an element *x* to some implicit counterproperty corresponding to a bipolar fuzzy set *B*. If *μ*_*B*_^*P*^(*x*) ≠ 0 and *μ*_*B*_^*N*^(*x*) = 0, it is the situation that *x* is regarded as having only positive satisfaction for *B*. If *μ*_*B*_^*P*^(*x*) = 0 and *μ*_*B*_^*N*^(*x*) ≠ 0, it is the situation that *x* does not satisfy the property of *B* but somewhat satisfies the counterproperty of *B*. It is possible for an element *x* to be such that *μ*_*B*_^*P*^(*x*) ≠ 0 and *μ*_*B*_^*N*^(*x*) ≠ 0 when the membership function of the property overlaps that of its counterpro perty over some portion of *X*.

For the sake of simplicity, we shall use the symbol *B* = (*μ*_*B*_^*P*^, *μ*_*B*_^*N*^) for the bipolar fuzzy set

(4) $$\begin{array}{c} B=\left\{\left(x,\mu_{B}^{P}\left(x\right),\mu_{B}^{N}\left(x\right)\right)\;|\;x\in X\right\}. \end{array}$$

A nice application of bipolar fuzzy concept is a political acceptation (map to [0, 1]) and nonacceptation (map to [−1, 0]).

**Definition 7 (see [3])**

A bipolar fuzzy relation *R* = (*μ*_*R*_^*P*^(*x*, *y*), *μ*_*R*_^*N*^(*x*, *y*)) in a universe *X* × *Y* (*R*(*X* → *Y*), for short) is a bipolar fuzzy set of the form

(5) $$\begin{array}{c} R=\left\{\langle \left(x,y\right),\mu_{R}^{P}\left(x,y\right),\mu_{R}^{N}\left(x,y\right)\rangle \;|\;\left(x,y\right)\in X\times Y\right\}, \end{array}$$

where *μ*_*R*_^*P*^ : *X* × *Y* → [0,1] and *μ*_*R*_^*N*^ : *X* × *Y* → [−1,0].

**Definition 8**

Let *R* be a bipolar fuzzy relation on universe *X*. Then *R* is called *a bipolar fuzzy equivalence relation* on *X* if it satisfies the following conditions:

- *R*  is bipolar fuzzy reflexive; that is, *R*(*x*, *x*) = (1, −1) for each *x* ∈ *X*;
- *R* is bipolar fuzzy symmetric; that is, *R*(*x*, *y*) = *R*(*y*, *x*) for any *x*, *y* ∈ *X*;
- *R* is bipolar fuzzy transitive; that is, *R*(*x*, *z*) ≥ ⋁_*y*_(*R*(*x*, *y*)∧*R*(*y*, *z*)).

**Definition 9**

Let *R* be a bipolar fuzzy relation on universe *X*. Then *R* is called *a bipolar fuzzy partial order relation* on *X* if it satisfies the following conditions:

- *R* is bipolar fuzzy reflexive; that is, *R*(*x*, *x*) = (1, −1) for each *x* ∈ *X*;
- *R* is bipolar fuzzy antisymmetric; that is, *R*(*x*, *y*) ≠ *R*(*y*, *x*) for any *x*, *y* ∈ *X*;
- *R* is bipolar fuzzy transitive; that is, *R*(*x*, *z*) ≥ ⋁_*y*_(*R*(*x*, *y*)∧*R*(*y*, *z*)).

**Definition 10**

Let *R* be a bipolar fuzzy relation on universe *X*. Then *R* is called *a bipolar fuzzy linear order relation* on *X* if it satisfies the following conditions:

- *R* is bipolar fuzzy partial relation;
- (*μ*_*R*_^*P*^∨*μ*_*R*^−1^_^*P*^)(*x*, *y*) > 0, (*μ*_*R*_^*N*^∧*μ*_*R*^−1^_^*N*^)(*x*, *y*) < 0 for all *x*, *y* ∈ *X*.

## 3. Cayley Bipolar Fuzzy Graphs

**Definition 11**

A *bipolar fuzzy digraph* of a digraph D* is a pair *D* = (*A*, *B*) where *A* = (*μ*_*A*_^*P*^, *μ*_*A*_^*N*^) is a bipolar fuzzy set in *V* and *B* = (*μ*_*B*_^*P*^, *μ*_*B*_^*N*^) is a bipolar fuzzy relation on *E* such that

(6) $$\begin{array}{c} \mu_{B}^{P}\left(xy\right)\leq \min\left(\mu_{A}^{P}\left(x\right),\mu_{A}^{P}\left(y\right)\right),\\ \mu_{B}^{N}\left(xy\right)\geq \max\left(\mu_{A}^{N}\left(x\right),\mu_{A}^{N}\left(y\right)\right) \end{array}$$

for all *xy* ∈ *E*. We note that *B* need not to be symmetric.

**Definition 12**

Let *D* be a bipolar fuzzy digraph. The indegree of a vertex *x* in *D* is defined by ind(*x*) = (ind_*μ*_^*P*^(*x*), ind_*μ*_^*N*^(*x*)), where ind_*μ*_^*P*^(*x*) = ∑_*y*≠*x*_ *μ*_*A*_^*P*^(*xy*) and ind_*μ*_^*N*^(*x*) = ∑_*y*≠*x*_ *μ*_*A*_^*N*^(*xy*). Similarly, the outdegree of a vertex *x* in *D* is defined by outd(*x*) = (outd_*μ*_^*P*^(*x*), outd_*μ*_^*N*^(*x*)), where outd_*μ*_^*P*^(*x*) = ∑_*y*≠*x*_ *μ*_*A*_^*P*^(*xy*) and outd_*μ*_^*N*^(*x*) = ∑_*y*≠*x*_ *μ*_*A*_^*N*^(*xy*). A bipolar fuzzy digraph in which each vertex has the same outdegree *r* is called *an outregular digraph* with index of outregularity *r*. In-regular digraphs are defined similarly.

**Definition 13**

Let (*V*, ∗) be a group and let *A* = (*μ*_*A*_^*P*^, *μ*_*A*_^*N*^) be the bipolar fuzzy subset of *V*. Then the bipolar fuzzy relation *R* defined on *V* by

(7) $$\begin{array}{c} R\left(x,y\right)=\left(\mu_{R}^{P}\left(x^{-1}y\right),\mu_{R}^{N}\left(\left(x^{-1}y\right)\right)\right),\;\forall x,y\in V, \end{array}$$

induces a bipolar fuzzy graph *G* = (*V*, *R*) called the Cayley bipolar fuzzy graph induced by the (*V*, ∗, *μ*_*R*_^*P*^, *μ*_*R*_^*N*^).

We now introduce Cayley bipolar fuzzy graphs and prove that all Cayley bipolar fuzzy graphs are regular.

**Definition 14**

Let (*V*, ∗) be a group and let *A* = (*μ*_*A*_^*P*^, *μ*_*A*_^*N*^) be a bipolar fuzzy subset of *V*. Then the bipolar fuzzy relation *R* on *V* defined by

(8) $$\begin{array}{c} R\left(x,y\right)=\left\{\mu_{R}^{P}\left(x^{-1}y\right),\mu_{R}^{N}\left(x^{-1}y\right)\;\forall x,y\in V\right\} \end{array}$$

induces a bipolar fuzzy graph *G* = (*V*, *R*), called the *Cayley bipolar fuzzy graph* induced by the (*V*, ∗, *A*).

**Example 15**

**Figure 1:**
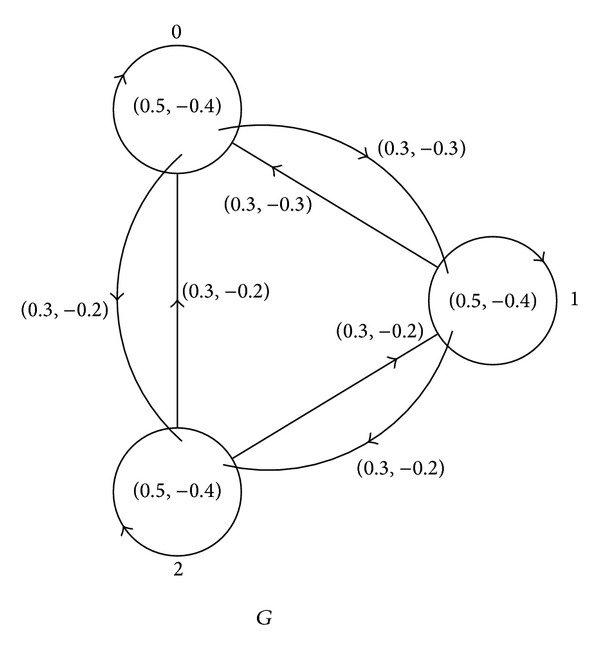
Cayley bipolar fuzzy graph.

Consider the group *Z*_3_ and take *V* = {0,1, 2}. Define *μ*_*A*_^*P*^ : *V* → [0,1] and *μ*_*A*_^*N*^ : *V* → [−1,0] by *μ*_*A*_^*P*^(0) = *μ*_*A*_^*P*^(1) = *μ*_*A*_^*P*^(2) = 0.5, *μ*_*A*_^*N*^(0) = *μ*_*A*_^*N*^(1) = *μ*_*A*_^*N*^(2) = −0.4. Then the Cayley bipolar fuzzy graph *G* = (*V*, *R*) induced by (*Z*_3_, +, *A*) is given by Table 1 and Figure 1.

We see that Cayley bipolar fuzzy graphs are actually bipolar fuzzy digraphs. Furthermore, the relation *R* in the above definition describes the strength of each directed edge. Let *G* denote a bipolar fuzzy graph *G* = (*V*, *R*) induced by the triple (*V*, ∗, *A*).

**Theorem 16**

The Cayley bipolar fuzzy graph *G* is vertex transitive.

**Proof**

Let *a*,  *b* ∈ *V*. Define *ψ* : *V* → *V* by *ψ*(*x*) = *ba* ^−1^ *x* for all *x* ∈ *V*. Clearly, *ψ* is a bijective map. For each *x*, *y* ∈ *V*,

(9) R(ψ(x),ψ(y))=(RμP(ψ(x),ψ(y)),    RμN(ψ(x),ψ(y))).Now  RμP(ψ(x),ψ(y))=RμP(ba−1x,ba−1y)=μAP((ba−1x)−1(ba−1x))=μAP(x−1y)=RμP(x,y),RμN(ψ(x),ψ(y))=RμN(ba−1x,ba−1y)=μAN((ba−1x)−1(ba−1x))=μAN(x−1y)=RμN(x,y).

Therefore, *R*(*ψ*(*x*), *ψ*(*y*)) = *R*(*x*, *y*). Hence *ψ* is an automorphism on *G*. Also *ψ*(*a*) = *b*. Hence *G* is vertex transitive.

**Theorem 17**

Every vertex transitive bipolar fuzzy graph is regular.

**Proof**

Let *G* = (*V*, *R*) be any vertex transitive bipolar fuzzy graph. Let *u*, *v* ∈ *V*. Then there is an automorphism *f* on *G* such that *f*(*u*) = *v*. Note that

(10) ind(u)=∑x∈VR(x,u)=∑x∈V(RμP(x,u),RμN(x,u))=∑x∈V(RμP(f(x),f(u)),RμN(f(x),f(u)))=∑x∈V(RμP(f(x),v),RμN(f(x),v))=∑x∈V(RμP(y,v),RμN(y,v))=ind(v),outd(u)=∑x∈VR(x,u)=∑x∈V(RμP(u,x),RμN(u,x))=∑x∈V(RμP(f(u),f(x)),RμN(f(u),f(x))) =∑x∈V(RμP(v,f(x)),RμN(v,f(x)))=∑x∈V(RμP(v,y),RμN(v,y)) =outd(v).

Hence *G* is regular.

**Theorem 18**

Cayley bipolar fuzzy graphs are regular.

**Proof**

Proof follows from Theorems 16 and 17.

**Theorem**

Let *G* = (*V*, *R*) denote bipolar fuzzy graph. Then bipolar fuzzy relation *R* is reflexive if and only if *μ*_*A*_^*P*^(1) = 1 and *μ*_*A*_^*N*^(1) = −1.

**Proof**

*R* is reflexive if and only if *R*(*x*, *x*) = (1, −1) for all *x* ∈ *V*. Now

(11) R(x,x)=(μAP(x−1x),μAN(x−1x))=(μAP(1),μAN(1)), ∀x∈V.

Hence *R* is reflexive if and only if *μ*_*A*_^*P*^(1) = 1 and *μ*_*A*_^*N*^(1) = −1.

**Theorem 20**

Let *G* = (*V*, *R*) denote bipolar fuzzy graph. Then bipolar fuzzy relation *R* is symmetric if and only if (*μ*_*A*_^*P*^(*x*), *μ*_*A*_^*N*^(*x*)) = (*μ*_*A*_^*P*^(*x* ^−1^), *μ*_*A*_^*N*^(*x* ^−1^)) for all *x* ∈ *V*.

**Proof**

Suppose that *R* is symmetric. Then for any *x* ∈ *V*,

(12) (μAP(x),μAN(x))=(μAP(x−1x2),μAN(x−1x2))=R(x,x2)=R(x2,x)  (since  R  is  symmetric)=(μAP((x2)−1x),μAN(x2)−1x)=μAP(x−2x),μAN(x−2x)=μAP(x−1),μAN(x−1).

Conversely, suppose that (*μ*_*A*_^*P*^(*x*), *μ*_*A*_^*N*^(*x*)) = (*μ*_*A*_^*P*^(*x* ^−1^), *μ*_*A*_^*N*^(*x* ^−1^)) for all *x* ∈ *V*. Then for all *x*, *y* ∈ *V*,

(13) R(x,y)=(μAP(x−1y),μAN(x−1y))=(μAP(y−1x),μAN(y−1x))=R(y,x).

Hence *R* is symmetric.

**Theorem 21**

A bipolar fuzzy relation *R* is antisymmetric if and only if {*x* : (*μ*_*A*_^*P*^(*x*), *μ*_*A*_^*N*^(*x*)) = (*μ*_*A*_^*P*^(*x* ^−1^), *μ*_*A*_^*N*^(*x* ^−1^)) = (1, −1).

**Definition 22**

Let (*S*, ∗) be a semigroup. Let *A* = (*μ*_*A*_^*P*^, *μ*_*A*_^*N*^) be a bipolar fuzzy subset of *S*. Then *A* is said to be a bipolar fuzzy subsemigroup of *S* if for all *x*, *y* ∈ *S*, *μ*_*B*_^*P*^(*xy*) ≥ *μ*_*A*_^*P*^(*x*)∧*μ*_*A*_^*P*^(*y*) and *μ*_*B*_^*N*^(*xy*) ≤ *μ*_*A*_^*N*^(*x*)∨*μ*_*A*_^*N*^(*y*).

**Theorem 23**

A bipolar fuzzy relation *R* is transitive if and only if *A* = (*μ*_*A*_^*P*^, *μ*_*A*_^*N*^) is a bipolar fuzzy subsemigroup of (*V*, ∗).

**Proof**

Suppose that *R* is transitive and let *x*, *y* ∈ *V*. Then *R* ^2^ ≤ *R*. Now for any *x* ∈ *V*, we have *R*(1, *x*) = (*μ*_*A*_^*P*^(*x*), *μ*_*A*_^*N*^(*x*)). This implies that {*R*(1, *z*)∧*R*(*z*, *xy*) : *z* ∈ *V*} = *R* ^2^(1, *xy*) ≤ *R*(1, *xy*). That is ∨{*μ*_*A*_^*P*^(*z*)∧*μ*_*A*_^*P*^(*z* ^−1^ *xy*) : *z* ∈ *V*} ≤ *μ*_*A*_^*P*^(*xy*) and ∧{*μ*_*A*_^*N*^(*z*)∨*μ*_*A*_^*N*^(*z* ^−1^ *xy*) : *z* ∈ *V*} ≥ *μ*_*A*_^*N*^(*xy*). Hence *μ*_*A*_^*P*^(*xy*) ≥ *μ*_*A*_^*P*^(*x*)∧*μ*_*A*_^*P*^(*y*) and *μ*_*A*_^*N*^(*xy*) ≤ *μ*_*A*_^*P*^(*x*)∨*μ*_*A*_^*N*^(*y*). Hence *A* = (*μ*_*A*_^*P*^, *μ*_*A*_^*N*^) is a bipolar fuzzy subsemigroup of (*V*, ∗).

Conversely, suppose that *A* = (*μ*_*A*_^*P*^, *μ*_*A*_^*N*^) is a bipolar fuzzy subsemigroup of (*V*, ∗). That is, for all *x*, *y* ∈ *Vμ*_*B*_^*P*^(*xy*) ≥ *μ*_*A*_^*P*^(*x*)∧*μ*_*A*_^*P*^(*y*) and *μ*_*B*_^*N*^(*xy*) ≤ *μ*_*A*_^*N*^(*x*)∨*μ*_*A*_^*N*^(*y*). Then for any *x*, *y* ∈ *V*,

(14) R2(x,y)=(RμP2(x,y),RμN2(x,y)),RμP2(x,y)=∨{RμP(x,z)∧RμP(z,y):z∈V}=∨{μAP(x−1z)∧μAP(z−1y):z∈V}≤μAP(x−1y)=RμP(x,y),RμN2(x,y)=∧{RμN(x,z)∨RμN(z,y):z∈V}=∧{μAN(x−1z)∨μAN(z−1y):z∈V}≥μAN(x−1y)=RμN(x,y).

Hence *R*_*μ*^*P*^_^2^(*x*, *y*) ≤ *R*_*μ*^*P*^_(*x*, *y*) and *R*_*μ*^*N*^_^2^(*x*, *y*) ≥ *R*_*μ*^*N*^_(*x*, *y*). Hence *R* is transitive.

We conclude that.

**Theorem 24**

A bipolar fuzzy relation *R* is a partial order if and only if *A* = (*μ*_*A*_^*P*^, *μ*_*A*_^*N*^) is a bipolar fuzzy subsemigroup of (*V*, ∗) satisfying

- *μ*_*A*_^*P*^(1) = 1 and *μ*_*A*_^*N*^(1) = −1,
- {*x* : (*μ*_*A*_^*P*^(*x*), *μ*_*A*_^*N*^(*x*)) = (*μ*_*A*_^*P*^(*x* ^−1^), *μ*_*A*_^*N*^(*x* ^−1^)) = {1, −1}}.

**Theorem 25**

A bipolar fuzzy relation *R* is a linear order if and only if (*μ*_*A*_^*P*^, *μ*_*A*_^*N*^) is a bipolar fuzzy subsemigroup of (*V*, ∗) satisfying

- *μ*_*A*_^*P*^(1) = 1 and *μ*_*A*_^*N*^(1) = −1,
- {*x* : (*μ*_*A*_^*P*^(*x*), *μ*_*A*_^*N*^(*x*)) = (*μ*_*A*_^*P*^(*x* ^−1^), *μ*_*A*_^*N*^(*x* ^−1^)) = {1, −1}},
- {*x* : *μ*_*A*_^*P*^(*x*)∨*μ*_*A*_^*P*^(*x* ^−1^) > 0, *μ*_*A*_^*N*^(*x*)∧*μ*_*A*_^*N*^(*x* ^−1^) < 0} = *V*.

**Proof**

Suppose *R* is a linear order. Then by Theorem 24, conditions (i), (ii), and (iii) are satisfied. For any *x* ∈ *V*, (*R*∨*R* ^−1^)(1, *x*) > 0. This implies that *R*(1, *x*)∨*R*(*x*, 1) > 0. Hence {*x* : *μ*_*A*_^*P*^(*x*)∨*μ*_*A*_^*P*^(*x* ^−1^) > 0, *μ*_*A*_^*N*^(*x*)∧*μ*_*A*_^*N*^(*x* ^−1^) < 0}.

Conversely, suppose that conditions (i), (ii), and (iii) hold. Then by Theorem 24, *R* is partial order. Now for any *x*, *y* ∈ *V*, we have (*x* ^−1^ *y*), (*y* ^−1^ *x*) ∈ *V*. Then by condition (iv), {*x* : *μ*_*A*_^*P*^(*x*)∨*μ*_*A*_^*P*^(*x* ^−1^) > 0, *μ*_*A*_^*N*^(*x*)∧*μ*_*A*_^*N*^(*x* ^−1^) < 0}. Therefore *R* is linear order.

**Theorem 26**

A bipolar fuzzy relation *R* is a equivalence relation if and only if (*μ*_*A*_^*P*^, *μ*_*A*_^*N*^) is a bipolar fuzzy subsemigroup of (*V*, ∗) satisfying

- *μ*_*A*_^*P*^(1) = 1 and *μ*_*A*_^*N*^(1) = −1,
- (*μ*_*A*_^*P*^(*x*), *μ*_*A*_^*N*^(*x*)) = (*μ*_*A*_^*P*^(*x* ^−1^), *μ*_*A*_^*N*^(*x* ^−1^)) for all *x* ∈ *V*.

**Theorem 27**

*G* is a Hasse diagram if and only if for any collection *x*_1_, *x*_2_, *x*_3_,…, *x*_*n*_ of vertices in *V* with *n* ≥ 2 and *μ*_*A*_^*P*^(*x*_*i*_) > 0, *μ*_*A*_^*N*^(*x*_*i*_) < 0, for *i* = 1,2, 3,…, *n*, we have, *μ*_*A*_^*P*^(*x*_1_ *x*_2_ ⋯ *x*_*n*_) = 0 and *μ*_*A*_^*N*^(*x*_1_ *x*_2_ ⋯ *x*_*n*_) = 0.

**Proof**

Suppose *G* is a Hasse diagram and let *x*_1_, *x*_2_,…, *x*_*n*_ be vertices in *V* with *n* ≥ 2 and *μ*_*A*_^*P*^(*x*_*i*_) > 0, *μ*_*A*_^*N*^(*x*_*i*_) < 0, for *i* = 1,2, 3,…, *n*. Then it is obvious that *R*(*x*_1_ *x*_2_ ⋯ *x*_*i*−1_, *x*_1_ *x*_2_ ⋯ *x*_*i*_) = (*μ*_*A*_^*P*^(*x*_*i*_), *μ*_*A*_^*N*^(*x*_*i*_)), for *i* = 1,2,…, *n*, where *x*_0_ = 1. Therefore (1, *x*_1_, *x*_1_ *x*_2_,…, *x*_1_ *x*_2_ ⋯ *x*_*n*_) is a path from 1 to *x*_1_ *x*_2_ ⋯ *x*_*n*_. Since *G* is a Hasse diagram, we have *R*(1, *x*_1_ *x*_2_ ⋯ *x*_*n*_) = 0. This implies that *μ*_*A*_^*P*^(*x*_1_ *x*_2_ ⋯ *x*_*n*_) = 0 and *μ*_*A*_^*N*^(*x*_1_ *x*_2_ ⋯ *x*_*n*_) = 0. Conversely, suppose that for any collection *x*_1_, *x*_2_,…*x*_*n*_ of vertices in *V* with *n* ≥ 2 and *μ*_*A*_^*P*^(*x*_*i*_) > 0, *μ*_*A*_^*N*^(*x*_*i*_) < 0, for *i* = 1,2, 3,…, *n*, we have, *μ*_*A*_^*P*^(*x*_1_ *x*_2_ ⋯ *x*_*n*_) = 0 and *μ*_*A*_^*N*^(*x*_1_ *x*_2_ ⋯ *x*_*n*_) = 0. Let (*x*_0_, *x*_1_, *x*_2_,…*x*_*n*_) be a path in *G* from *x*_0_ to *x*_*n*_ with *n* ≥ 2. Then *R*(*x*_*i*−1_, *x*_*i*_) > 0, for *i* = 1,2,…, *n*. Therefore *μ*_*A*_^*P*^(*x*_*i*−1_^−1^ *x*_*i*_) > 0, *μ*_*A*_^*N*^(*x*_*i*−1_^−1^ *x*_*i*_) < 0, for *i* = 1,2,…, *n*. Now consider the elements *x*_0_^−1^ *x*_1_, *x*_1_^−1^ *x*_2_,…, *x*_*n*−1_^−1^ *x*_*n*_ in *V*. Then by assumption *μ*_*A*_^*P*^(*x*_0_^−1^ *x*_1_ *x*_1_^−1^ *x*_2_ ⋯ *x*_*n*−1_^−1^ *x*_*n*_) = 0 and *μ*_*A*_^*N*^(*x*_0_^−1^ *x*_1_ *x*_1_^−1^ *x*_2_ ⋯ *x*_*n*−1_^−1^ *x*_*n*_) = 0. That is, *μ*_*A*_^*P*^(*x*_0_^−1^ *x*_*n*_) = 0 and *μ*_*A*_^*N*^(*x*_0_^−1^ *x*_*n*_) = 0. Hence, *R*(*x*_0_, *x*_*n*_) = 0. Thus *G* is a Hasse diagram.

Let *G* = (*V*, *R*) be any bipolar fuzzy graph; then *G* is connected (weakly connected, semiconnected, locally connected, or quasi-connected) if and only if the induce fuzzy graph (*V*, *R*_0_^+^) is connected (weakly connected, semiconnected, locally connected, or quasi-connected).

**Definition 28**

Let (*S*, ∗) be a semigroup and let *A* = (*μ*_*A*_^*P*^, *μ*_*A*_^*N*^) be a bipolar fuzzy subset of *S*. Then the subsemigroup generated by *A* is the meeting of all bipolar fuzzy subsemigroups of *S* which contains *A*. It is denoted by 〈*A*〉.

**Lemma 29**

Let (*S*, ∗) be a semigroup and *A* = (*μ*_*A*_^*P*^, *μ*_*A*_^*N*^) be a bipolar fuzzy subset of *S*. Then bipolar fuzzy subset 〈*A*〉 is precisely given by 〈*μ*_*A*_^*P*^〉(*x*) = ∨{*μ*_*A*_^*P*^(*x*_1_)∧*μ*_*A*_^*P*^(*x*_2_)∧⋯∧*μ*_*A*_^*P*^(*x*_*n*_) : *x* = *x*_1_ *x*_2_ ⋯ *x*_*n*_ with  *μ*_*A*_^*P*^(*x*_*i*_) > 0 for *i* = 1,2,…, *n*}, 〈*μ*_*A*_^*N*^〉(*x*) = ∧{*μ*_*A*_^*N*^(*x*_1_)∨*μ*_*A*_^*N*^(*x*_2_)∨⋯∨*μ*_*A*_^*N*^(*x*_*n*_) : *x* = *x*_1_ *x*_2_ ⋯ *x*_*n*_ with *μ*_*A*_^*N*^(*x*_*i*_) < 0 *fori* = 1,2,…, *n*} for any *x* ∈ *S*.

**Proof**

Let $A^{\prime }=({\overset{´}{\mu }}_{A}^{P},{\overset{´}{\mu }}_{A}^{N})$ be a bipolar fuzzy subset of *S* defined by ${\overset{´}{\mu }}_{A}^{P}(x)=\vee \{\mu_{A}^{P}(x_{1})\wedge \mu_{A}^{P}(x_{2})\wedge \cdots \wedge \mu_{A}^{P}(x_{n}):x=x_{1}x_{2}\cdots x_{n}$ with *μ*_*A*_^*P*^(*x*_*i*_) > 0 for *i* = 1,2,…, *n*}, ${\overset{´}{\mu }}_{A}^{N}(x)=\wedge \{\mu_{A}^{N}(x_{1})\vee \mu_{A}^{N}(x_{2})\vee \cdots \vee \mu_{A}^{N}(x_{n}):x=x_{1}x_{2}\cdots x_{n}$ with *μ*_*A*_^*N*^(*x*_*i*_) < 0 for *i* = 1,2,…, *n*}, for any *x* ∈ *S*. Let *x*, *y* ∈ *S*. If *μ*_*A*_^*P*^(*x*) = 0 or *μ*_*A*_^*P*^(*y*) = 0, then *μ*_*A*_^*P*^(*x*)∧*μ*_*A*_^*P*^(*y*) = 0 and *μ*_*A*_^*N*^(*x*) = 0 or *μ*_*A*_^*N*^(*y*) = 0, and then *μ*_*A*_^*N*^(*x*)∨*μ*_*A*_^*N*^(*y*) = 0. Therefore, ${\overset{´}{\mu }}_{B}^{P}(xy)\geq \mu_{A}^{P}(x)\wedge \mu_{A}^{P}(y)$ and ${\overset{´}{\mu }}_{B}^{N}(xy)\leq \mu_{A}^{N}(x)\vee \mu_{A}^{N}(y)$. Again, if *μ*_*A*_^*P*^(*x*) ≠ 0, *μ*_*A*_^*N*^(*x*) ≠ 0, then by definition of ${\overset{´}{\mu }}_{A}^{P}(x)$ and ${\overset{´}{\mu }}_{A}^{N}(x)$, we have ${\overset{´}{\mu }}_{B}^{P}(xy)\geq \mu_{A}^{P}(x)\wedge \mu_{A}^{P}(y)$ and ${\overset{´}{\mu }}_{B}^{N}(xy)\leq \mu_{A}^{N}(x)\vee \mu_{A}^{N}(y)$. Hence $\left({\overset{´}{\mu }}_{A}^{P},{\overset{´}{\mu }}_{A}^{N}\right)$ is a bipolar fuzzy subsemigroup of *S* containing (*μ*_*A*_^*P*^, *μ*_*A*_^*N*^). Now let *L* be any bipolar fuzzy subsemigroup of *S* containing (*μ*_*A*_^*P*^, *μ*_*A*_^*N*^). Then for any *x* ∈ *S* with *x* = *x*_1_ *x*_2_ ⋯ *x*_*n*_ with *μ*_*A*_^*P*^(*x*_*i*_) > 0, *μ*_*A*_^*N*^(*x*_*i*_) < 0, for *i* = 1,2,…, *n*, we have *μ*_*L*_^*P*^(*x*_*i*_) ≥ *μ*_*L*_^*P*^(*x*_1_)∧*μ*_*L*_^*P*^(*x*_2_)∧⋯∧*μ*_*L*_^*P*^(*x*_*n*_) ≥ *μ*_*A*_^*P*^(*x*_1_)∧*μ*_*A*_^*P*^(*x*_2_)∧⋯∧*μ*_*A*_^*P*^(*x*_*n*_) and *μ*_*L*_^*N*^(*x*_*i*_) ≥ *μ*_*L*_^*N*^(*x*_1_)∧*μ*_*L*_^*N*^(*x*_2_)∧⋯∧*μ*_*L*_^*N*^(*x*_*n*_) ≥ *μ*_*A*_^*N*^(*x*_1_)∧*μ*_*A*_^*N*^(*x*_2_)∧⋯∧*μ*_*A*_^*N*^(*x*_*n*_). Thus *μ*_*L*_^*P*^(*x*)≥∨{*μ*_*A*_^*P*^(*x*_1_)∧*μ*_*A*_^*P*^(*x*_2_)∧⋯∧*μ*_*A*_^*P*^(*x*_*n*_) : *x* = *x*_1_ *x*_2_ ⋯ *x*_*n*_ with *μ*_*A*_^*P*^(*x*_*i*_) > 0 for *i* = 1,2,…, *n*}, *μ*_*L*_^*N*^(*x*)≤∧{*μ*_*A*_^*N*^(*x*_1_)∨*μ*_*A*_^*N*^(*x*_2_)∨⋯∨*μ*_*A*_^*N*^(*x*_*n*_) : *x* = *x*_1_ *x*_2_ ⋯ *x*_*n*_ with *μ*_*A*_^*N*^(*x*_*i*_) < 0 for *i* = 1,2,…, *n*}, for any *x* ∈ *S*. Hence $\mu_{L}^{P}(x)\geq {\overset{´}{\mu }}_{A}^{P}(x),\mu_{L}^{N}(x)\leq {\overset{´}{\mu }}_{A}^{N}(x)$, for all *x* ∈ *S*. Thus ${\overset{´}{\mu }}_{A}^{P}(x)\leq \mu_{L}^{P}(x)$, ${\overset{´}{\mu }}_{A}^{N}(x)\geq \mu_{A}^{P}(x)$. Thus $A^{\prime }=({\overset{´}{\mu }}_{A}^{P},{\overset{´}{\mu }}_{A}^{N})$ is the meeting of all bipolar fuzzy subsemigroups containing (*μ*_*A*_^*P*^, *μ*_*A*_^*N*^).

**Theorem 30**

Let (*S*, ∗) be a semigroup and *A* = (*μ*_*A*_^*P*^, *μ*_*A*_^*N*^) be a bipolar fuzzy subset of *S*. Then for any *α* ∈ [0,1], (〈*μ*_*α*_^*P*^〉, 〈*μ*_*α*_^*N*^〉) = (〈*μ* ^*P*^〉_*α*_, 〈*μ* ^*N*^〉_*α*_) and (〈(*μ* ^+^)_*α*_^*P*^〉, 〈(*μ* ^+^)_*α*_^*N*^〉) = (〈*μ* ^*P*^〉_*α*_^+^, 〈*μ* ^*N*^〉_*α*_^+^), where (〈*μ*_*α*_^*P*^〉, 〈*μ*_*α*_^*N*^〉) denotes the subsemigroup generated by (*μ*_*α*_^*P*^, *μ*_*α*_^*N*^) and 〈(*μ* ^*P*^, *μ* ^*N*^)〉 denotes bipolar fuzzy subsemigroup generated by (*μ* ^*P*^, *μ* ^*N*^).

**Proof**

(15) x∈(〈μP〉α,〈μN〉α)⇔there  exists  x1,x2,…,xn  in(μαP,μαN)  such  that  x=x1x2⋯xn⇔there  exists  x1,x2,…,xn  in  S  such  that  μP(xi)≥α,μN(xi)≤α,  ∀i=1,2,…,n,x=x1x2⋯xn⇔〈μP〉(x)≥α,〈μN〉(x)≤α⇔x∈〈μP〉α,x∈〈μN〉α.

Therefore (〈*μ*_*α*_^*P*^〉, 〈*μ*_*α*_^*N*^〉) = (〈*μ* ^*P*^〉_*α*_, 〈*μ* ^*N*^〉_*α*_). Similarly, we have (〈(*μ* ^+^)_*α*_^*P*^〉, 〈(*μ* ^+^)_*α*_^*N*^〉) = (〈*μ* ^*P*^〉_*α*_^+^, 〈*μ* ^*N*^〉_*α*_^+^).

**Remark 31**

Let (*S*, ∗) be a semigroup and *A* = (*μ*_*A*_^*P*^, *μ*_*A*_^*N*^) be a bipolar fuzzy subset of *S*. Then by Theorem 30, we have 〈supp⁡(*A*) = *A* ^+^〉 = supp⁡〈*A*〉.

Let *G* denote the Cayley bipolar fuzzy graphs *G* = (*V*, *R*) induced by (*V*, ∗, *μ* ^*P*^, *μ* ^*N*^). Then we have the following results.

**Theorem 32**

Let *A* be any subset of *V*′ and *G*′ = (*V*′, *R*′) be the Cayley graph induced by (*V*′, ∗, *A*). Then *G*′ is connected if and only if 〈*A*〉⊇*V* − *v*_1_.

**Theorem 33**

*G* is connected if and only if  supp⁡〈*A*〉⊇*V* − *v*_1_.

**Theorem 34**

Let *A* be any subset of a set *V*′ and let *G*′ = (*V*′, *R*′) be the Cayley graph induced by the triplet (*V*′, ∗, *A*). Then *G*′ is weakly connected if and only if 〈*A* ∪ *A* ^−1^〉⊇*V* − *v*_1_, where *A* ^−1^ = {*x* ^−1^ : *x* ∈ *A*}.

**Definition 35**

Let (*S*, ∗) be a group and let *A* be a bipolar fuzzy subset of *S*. Then we define *A* ^−1^ as bipolar fuzzy subset of *S* given by *A* ^−1^(*x*) = *A*(*x* ^−1^) for all *x* ∈ *S*.

**Theorem 36**

*G* is weakly connected if and only if supp⁡(〈*A* ∪ *A* ^−1^〉)⊇*V* − *v*_1_.

**Proof**

(16) G  is  weakly  connected⇔(V,R0+)is  weakly  connected⇔〈A0+∪(A0+)−1〉⊇V−v1⇔〈supp⁡(A)∪supp⁡(A)−1〉⊇V−v1⇔supp⁡〈A∪(A)−1〉⊇V−v1⇔supp⁡〈A∪A−1〉⊇V−v1.

**Theorem 37**

Let *A* be any subset of a set *V*′ and let *G*′ = (*V*′, *R*′) be the Cayley graph induced by the triplet (*V*′, ∗, *A*). Then *G*′ is semiconnected if and only if 〈*A*〉 ∪ 〈*A* ^−1^〉⊇*V* − *v*_1_, where *A* ^−1^ = {*x* ^−1^ : *x* ∈ *A*}.

**Theorem 38**

*G* is semi-connected if and only if supp⁡(〈*A*〉 ∪ 〈*A* ^−1^〉)⊇*V* − *v*_1_.

**Proof**

(17) G  is  semiconnected⇔(V,R0+)is  semi  connected⇔〈A0+〉∪〈(A0+)−1〉⊇V−v1⇔〈supp⁡(A)〉∪〈supp⁡(A)−1〉⊇V−v1⇔supp⁡〈A〉∪〈(A)−1〉⊇V−v1⇔supp⁡(〈A〉∪〈A−1〉)⊇V−v1.

**Theorem 39**

Let *G*′ = (*V*′, *R*′) be the Cayley graph induced by the triplet (*V*′, ∗, *A*). Then *G*′ is locally connected if and only if 〈*A*〉 = 〈*A* ^−1^〉, where *A* ^−1^ = (*x* ^−1^ : *x* ∈ *A*).

**Theorem 40**

Let *G* is locally connected if and only if supp⁡(〈*A*〉) = supp⁡(〈*A* ^−1^〉).

**Proof**

(18) G  is  locally  connected⇔(V,R0+)  is  locally  connected⇔〈A0+〉=〈(A0+)−1〉⇔〈supp⁡(A)〉=〈supp⁡(A)−1〉⇔supp⁡〈A〉=supp⁡〈A−1〉.

**Theorem 41**

Let *G*′ = (*V*′, *R*′) be the Cayley graph induced by the triplet (*V*′, ∗, *A*), where *V*′ is finite. Then *G*′ is quasi-connected if and only if it is connected.

**Theorem 42**

A finite Cayley bipolar fuzzy graph *G* is quasi-connected if and only if it is connected.

**Proof**

(19) G  is  quasi-connected⇔(V,R0+)  is  quasi-connected⇔(V,R0+)  is  connected⇔G  is  connected.

**Definition 43**

The *μ* ^*P*^ strength of a path *P* = *v*_1_, *v*_2_,…, *v*_*n*_ is defined as min⁡(*μ*_2_^*P*^(*v*_*i*_, *v*_*j*_)) for all *i* and *j* and is denoted by *S*_*μ*_^*P*^. The *μ* ^*N*^ strength of a path *P* = *v*_1_, *v*_2_,…, *v*_*n*_ is defined as max⁡(*μ*_2_^*N*^(*v*_*i*_, *v*_*j*_)) for all *i* and *j* and is denoted by *S*_*μ*_^*N*^.

**Definition 44**

Let *G* = (*V*, *μ* ^*P*^, *μ* ^*N*^) be a bipolar fuzzy graph. Then *G* is said to be

1. *α-connected* if for every pair of vertices *x*, *y* ∈ *G*, there is a path *P* from *x* to *y* such that strength (*P*) ≥ *α*,
2. *weakly* *α-connected* if a bipolar fuzzy graph (*V*, *R*∨*R* ^−1^) is *α*-connected,
3. *semi*-*α-connected* if for every *x*, *y* ∈ *V*, there is a path of strength greater than or equal to *α* from *x* to *y* or from *y* to *x* in *G*,
4. *locally* *α-connected* if for every pair of vertices *x* and *y*, there is a path *P* of strength greater than or equal to *α* from *x* to *y* whenever there is a path *P*′ of strength greater than or equal to *α* from *y* to *x*,
5. *quasi*-*α-connected* if for every pair *x*, *y* ∈ *V*, there is some *z* ∈ *V* such that there is directed path from *z* to *x* of strength greater than or equal to *α* and there is a directed path from *z* to *y* of strength greater than or equal to *α*.

**Remark 45**

Let *G* = (*V*, *R*) be any bipolar fuzzy graph; then *G* is *α*-connected (weakly *α*-connected, semi *α*-connected, locally *α*-connected or quasi *α*-connected) if and only if the induce fuzzy graph (*V*, *R*_0_^+^) is connected (weakly connected, semiconnected, locally connected, or quasi-connected).

Let *G* denote the Cayley bipolar fuzzy graphs *G* = (*V*, *R*) induced by (*V*, ∗, *μ* ^*P*^, *μ* ^*N*^). Also for any *α* ∈ [−1,1], we have the following results.

**Theorem 46**

*G* is *α*-connected if and only if 〈*A*〉_*α*_⊇*V* − *v*_1_.

**Proof**

(20) G  is  connected⇔(V,Rα)  is  connected⇔〈Aα〉⊇V−v1⇔〈A〉α⊇V−v1.

**Theorem 47**

*G* is weakly *α*-connected if and only if 〈*A*∪*A* ^−1^〉_*α*_⊇*V* − *v*_1_.

**Proof**

(21) G  is  weakly  connected⇔(V,Rα)  is  weakly  connected⇔〈Aα∪(Aα)−1〉⊇V−v1⇔〈(A∪A−1)α〉⊇V−v1⇔〈A∪(A)−1〉α⊇V−v1.

**Theorem 48**

*G* is semi-*α*-connected if and only if (〈*A*〉_*α*_ ∪ 〈*A* ^−1^〉_*α*_)⊇*V* − *v*_1_.

**Theorem 49**

Let *G* be locally *α*-connected if and only if 〈*A*〉_*α*_ = 〈*A*_*α*_^−1^〉.

**Theorem 50**

A finite Cayley bipolar fuzzy graph *G* is quasi-*α*-connected if and only if it is *α*-connected.

## 4. Conclusions

Fuzzy graph theory is finding an increasing number of applications in modeling real time systems where the level of information inherent in the system varies with different levels of precision. Fuzzy models are becoming useful because of their aim of reducing the differences between the traditional numerical models used in engineering and sciences and the symbolic models used in expert systems. A bipolar fuzzy set is a generalization of the notion of a fuzzy set. We have introduced the notion of Cayley bipolar fuzzy graphs in this paper. The natural extension of this research work is application of bipolar fuzzy digraphs in the area of soft computing including neural networks, decision making, and geographical information systems.

## Figures and Tables

**Table 1:** *R*(*a*, *b*) for Cayley bipolar fuzzy graph.

*a*	0	0	0	1	1	1	2	2	2
*b*	0	1	2	0	1	2	0	1	2
(−*a*) + *b*	0	1	2	2	0	1	1	2	0
*R*(*a*, *b*)	(0.5, −0.4)	(0.3, −0.2)	(0.3, −0.2)	(0.3, −0.2)	(0.5, −0.4)	(0.3, −0.2)	(0.3, −0.2)	(0.3, −0.2)	(0.5, −0.4)
